# Reef fish assemblages on *Acropora* patches in the Colombian Caribbean: resistance to reef degradation

**DOI:** 10.7717/peerj.21744

**Published:** 2026-10-05

**Authors:** Rocio García-Urueña, Silvia L. Sierra-Escrigas, Arturo Acero P

**Affiliations:** 1 Grupo de Investigación Ecología y Diversidad de Algas Marinas y Arrecifes Coralinos, Universidad del Magdalena, Santa Marta, Colombia; 2 Instituto de Estudios en Ciencias del Mar, CECIMAR, Universidad Nacional de Colombia sede Caribe, Santa Marta, Colombia

**Keywords:** Acropora corals, Reef fishes, Damselfishes, Wrasses

## Abstract

Coral reefs are crucial habitats for many fish species because they serve as feeding and shelter sites. However, due to their sensitivity to environmental changes, reef degradation has led to a drastic decline in fish and reef assemblages, which has also been reflected in associated economic activities. The loss of branching corals *Acropora palmata* and *A. cervicornis* has reached up to 90% in the Colombian Caribbean, resulting in a reduction in the abundance and diversity of associated fish populations. During two time periods (2011–2012 and 2023–2024) across the Colombian Caribbean, fish associated with *Acropora* formations were evaluated in relation to patch size and health status of these coral species. The best conserved patches of *A. palmata* were found in the Tayrona National Natural Park (TNNP) and of *A. cervicornis* in the reefs of the Urabá Gulf. A total of 113 fish species from 34 families were associated with *A. palmata*, with the most represented families being Labridae, Haemulidae, Pomacentridae, and Serranidae. *Acanthurus coeruleus, A. tractus, Chaetodon ocellatus, Haemulon flavolineatum, Scarus iseri*, *Sparisoma viride*, * Thalassoma bifasciatum, Abudefduf saxatilis*, * Microspathodon chrysurus*, *Stegastes adustus*, and * Canthigaster rostrata* were the species common to all reef locations. In *A. cervicornis* formations, 78 species belonging to 25 families were recorded, with Labridae and Pomacentridae as the most abundant. *Stegastes planifrons*, * Cephalopholis cruentata,* and *Hypoplectrus puella* were the species common to all locations. The highest number of fish species was found in the TNNP (85 for *A. palmata*, 55 for *A. cervicornis*). Although the status is critical throughout the Caribbean, further aggravated by the 2023–2024 bleaching event which caused *A. palmata* formations to practically disappear from two localities, this structural loss has not yet been reflected in the associated ichthyofauna. These results suggest that the fish assemblages linked to *Acropora* corals in the Colombian Caribbean remain relatively stable; however, it is unclear for how long they will persist.

## Introduction

The structural complexity provided by reef systems is crucial for the life cycles of numerous species, including fish (Hixon & Beets, 1993; Johnson et al., 2011; Graham & Nash, 2013) and crustaceans (Brown & Edmunds, 2013). A large number of fish species maintain strong relationships with reefs, relying on them for protection and feeding (Jones et al., 2004; Pratchett et al., 2012), leading to significant competitive interactions (Garpe et al., 2006; Cabaitan, Gómez & Aliño, 2008) and contributing to habitat quality, influencing community structure and fish abundance (Hixon & Beets, 1993; Findley & Findley, 2001; Graham & Nash, 2013).

Habitat quality has been linked to colony size and volume, as space availability is positively correlated with fish abundance and species diversity (Holbrook, Brooks & Schmitt, 2002; Hobbs & Munday, 2004; Schiemer, Niedermüller & Herler, 2009). However, shallow habitats and particularly coral ecosystems have experienced significant loss since the late 1970s (Gardner et al., 2003; Pandolfi et al., 2005; Aronson & Precht, 2006; Pandolfi & Jackson, 2006; Hoegh-Guldberg et al., 2007), threatening ecosystem functioning and numerous economic activities, as well as the abundance of coral reef fish (Gardner et al., 2003; Jones et al., 2004; Munday, 2004; Wilson et al., 2006; Coker, Graham & Pratchett, 2012).

Branched coral species have been demonstrated to be strong predictors of fish communities, as well as predictors of the survival and reproductive success of their populations (Jones, 1988; Holbrook, Forrester & Schmitt, 2000; Holbrook, Brooks & Schmitt, 2002; Pereira et al., 2012; Brooker et al., 2013; Leal, Pereira & De Araujo, 2013; Hernández-Delgado & Laureano, 2024). Thus, important reef-building species such as the elkhorn coral, *Acropora palmata*, and the staghorn coral, *A. cervicornis*, which used to dominate shallow sites, have not been exempt from this decline and have practically disappeared from many Caribbean regions due to factors such as the white band disease, predation, hurricanes, bleaching, and human-induced damage (Aronson & Precht, 2001; Gardner et al., 2003; Manzello et al., 2025). The situation in Colombia is not better, and these two coral species are listed as Endangered and Critically Endangered in the Colombian Red Book of Marine Invertebrates (García-Urueña, Alvarado-Chacón & Sanjuan-Muñoz, 2022; García-Urueña, Alvarado-Chacón & Acosta, 2022).

Environmental variability has had significant implications, including direct effects on the abundance and diversity of associated communities, through the reduction of available resources (Lemoine & Valentine, 2012), as well as the modification of local hydrodynamic environments, coastal geology, and fishing and tourism economies (Rao et al., 2015; Elliff & Silva, 2017). Naturally, the associated fish fauna has been affected, resulting in changes in its structure (Jones et al., 2004; Wilson et al., 2006). In this context, this study examined fish assemblages linked to *Acropora* patches during two periods separated by nearly a decade, in order to corroborate if *Acropora* structures are directly related to the use by reef fish species, as well as the variation of fish assemblages between localities along the Colombian Caribbean.

## Materials & Methods

*Acropora palmata* and *A. cervicornis* formations were surveyed and georeferenced across the continental and insular Colombian Caribbean during 2011–2012 and 2023–2024 (Fig. 1, Table S1). Observed colonies were classified into three categories based on number of colonies and patch size: scattered (1–2 isolated small colonies, larger than 1 m^2^), moderate (3–9 colonies separated by <10 m and occurring in an area less than 100 m^2^), and large (continuous formation extending >20 m, occupying an area larger than 100 m^2^). In 2023–2024, several new patches of both species were included; however, some formations that were visited during the 2011–2012 campaign had disappeared from some localities (Table S1).

**Figure 1:**
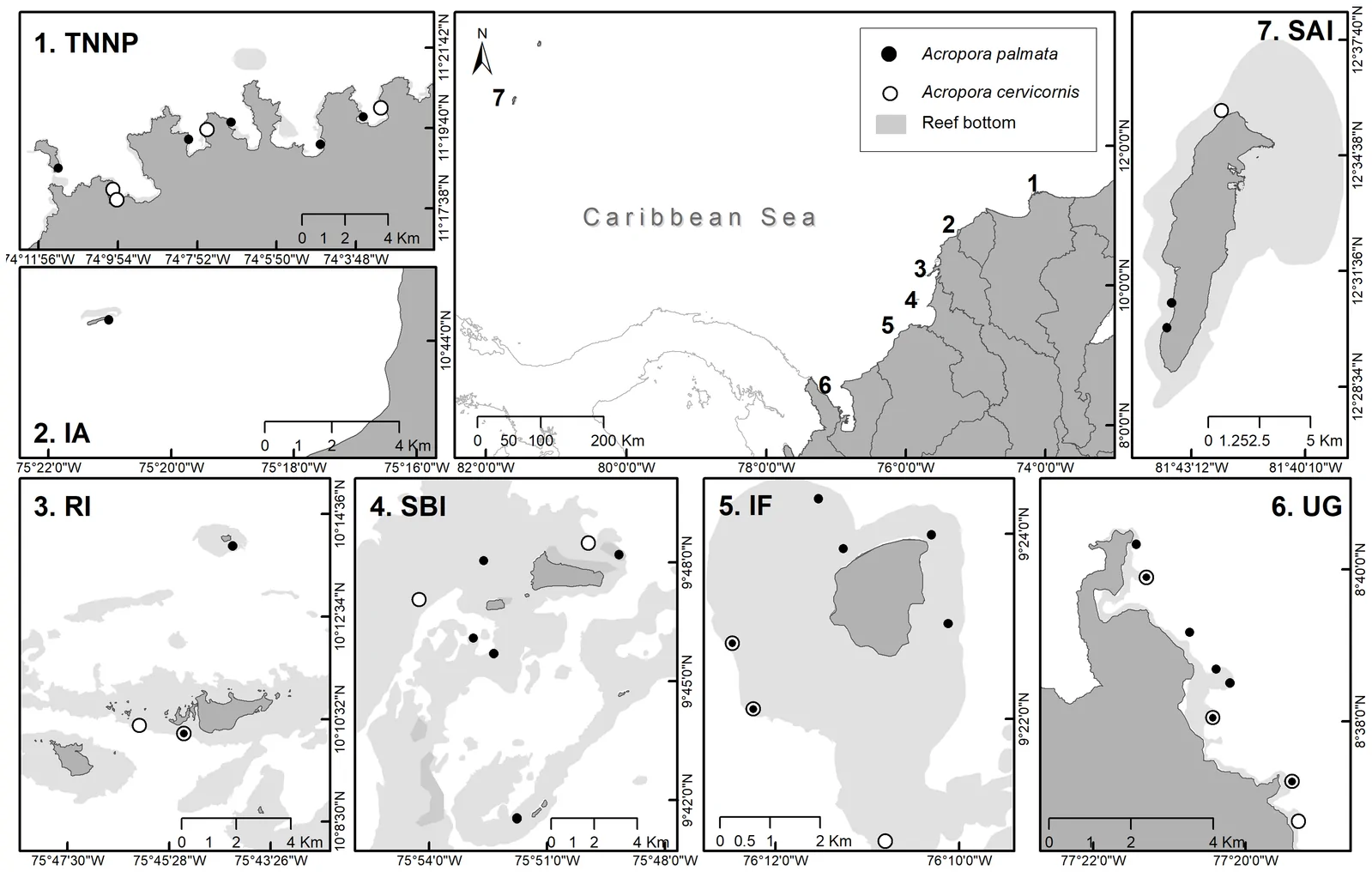
*Acropora palmata* and *A. cervicornis* localities surveyed along the Colombian Caribbean. Tayrona National Natural Park (TNNP), Isla Arena (IA), Rosario Islands (RI), San Bernardo Islands (SBI), Urabá Gulf (UG), Isla Fuerte (IF), and San Andrés Islands (SAI).

Fish assemblage structure associated with *Acropora* patches was characterized by 44 surveys at 29 sites from seven localities for elkhorn coral, and 23 surveys at 16 sites from six localities for staghorn coral (Table S1) were studied. In all visits SCUBA surveys were conducted for 30 min in each alive *Acropora* patch by looking around the colonies, identifying and recording all associated fish species observed. Colony depth ranged between 3 and 12 m, and all surveys were conducted between 8:00 and 14:00 h. All observations were made by the same experienced researcher to ensure consistency. Fish species were assigned to trophic level functional group (sessile invertivore, mobile benthic invertivore, piscivore, planktivore, omnivore, herbivore-detritivore, macroalgal feeder, and cleaner); position in water column was categorized in three: benthic, benthopelagic, and pelagic (Hernández-Delgado & Laureano, 2024). The study was carried out with the scientific research and biological diversity permit PIC No 004-2021 granted by National Natural Parks of Colombia.

To examine the relationship between the status of *Acropora* formations and their associated fish, we evaluated temporal changes in species richness and assemblage composition of fishes in each locality. For each *Acropora* species, fish species richness was assessed at different locations using an analysis of variance (ANOVA). For elkhorn coral, data were log-transformed to meet the assumptions of normality and homogeneity of variance. The Tukey’s pairwise test was used to compare significance between locations. Statistical analyses were made using SPSS Statistics version 22.0 and Statgraphics XVII software.

The structure of the fish assemblages associated with each *Acropora* species at each reef was analyzed using the Sørensen coefficient (Bray–Curtis similarity with binary data). A permutational multivariate analysis of variance (PERMANOVA) was performed, with patch size as a factor of each patch (scattered colonies, moderate patch, or large patch). In addition, a similarity percentages (SIMPER) analysis was conducted using the binary matrix of the transformed data to determine the contribution of fish species to spatial differences. Statistical analyses were conducted using PRIMER v7 (Clark & Gorley, 2015).

## Results

### Status of *Acropora palmata* and *A. cervicornis* populations during 2023–2024

*Acropora palmata* formations showed mortality associated with bleaching in two locations during the 2023–2024 El Niño event. For San Bernardo Islands, bleaching began between July and August 2023 in moderate sized patches; by October 2023, mortality has reached 100%. In Isla Arena, a relatively large formation existed between 1 and 3 m deep, but the entire formation bleached during the 2023 temperature increase, with mortality close to 50%. By 2024, the colonies suffered bleaching again. The hybrid *A. prolifera,* found only in this locality, was also completely bleached.

In the Rosario Islands only scattered colonies remained alive at depths greater than four meters. In the Urabá Gulf living colonies were observed between 2 and 10 m deep, generally scattered and in good condition. In San Andrés small and scattered colonies were found in the protected western side of the island; the northern patches, showed a marked loss by 2024, with mortality of more than 90%. The most important formation in Isla Fuerte, known as Punta El Inglés, showed 100% colony mortality between 1 and 3 m deep. However, numerous moderate patches of this species remain alive. The most important formations in the continental Colombian Caribbean are found in the Tayrona National Natural Park (TNNP), with the largest areas in Chengue and Cinto bays and numerous patches of moderate size in Isla Aguja, Gayraca, and Nenguange bays (Fig. 2A).

**Figure 2:**
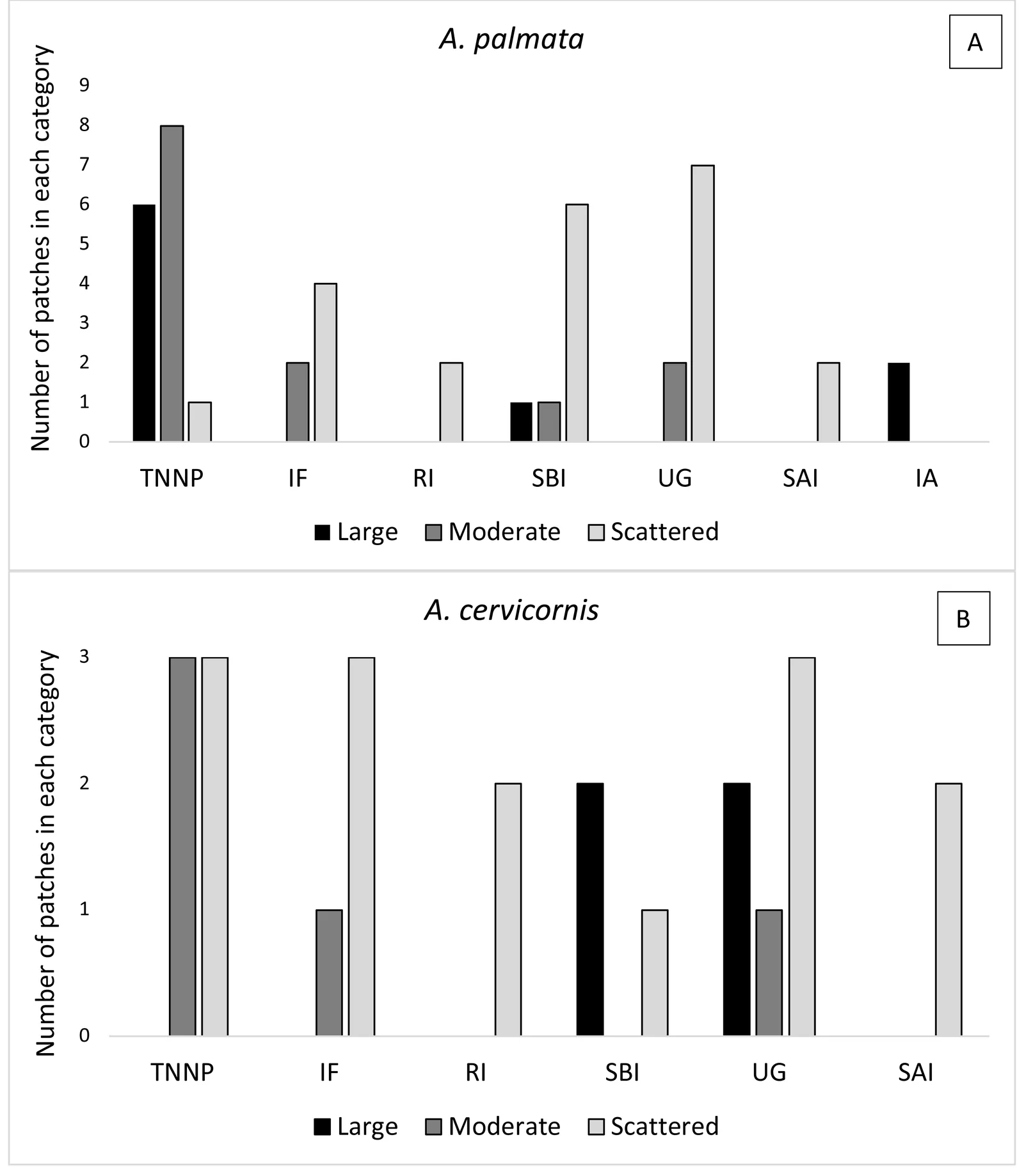
Patch size distribution of *Acropora palmata* (A), *Acropora cervicornis* (B) in the Colombian Caribbean (2023–2024). Tayrona National Natural Park (TNNP), Isla Fuerte (IF), Rosario Islands (RI), San Bernardo Islands (SBI), Urabá Gulf (UG), San Andrés islands (SAI), Isla Arena (IA).

In general, *A. cervicornis* is in critical condition throughout the Colombian Caribbean. In the Urabá Gulf, El Aguacate reef contained the healthiest and largest formations, but our observations indicate a marked mortality in the last years. In the TNNP only remnants of small patches were found; however, new patches were discovered in Concha and Chengue bays. In Rosario Islands, only scattered colonies were found in Caribarú and Isla Tesoro. In San Bernardo Islands, only small, scattered colonies remain, although in 2023 a large patch of 472.9 m^2^ was found far from the coast. In Isla Arena, the species disappeared completely. In Isla Fuerte, moderate patches were found in areas far from shore at depths greater than 10 m. The largest staghorn coral formation in the entire Colombian Caribbean detected previously at Cayo Bolívar (a small island part of the San Andrés Archipelago), was found completely dead in 2024. In San Andrés Island this species persists only as small, scattered colonies in Bajo Bonito reef (Fig. 2B).

For both species, the number of sampled sites increased between 2023 and 2024; however, for *A. cervicornis* the new patches were generally scattered, and widespread mortality was evident in those previously studied. Nevertheless, well-preserved and extensive patches of staghorn coral were found in San Bernardo Islands (approximately 462.9 m^2^) and in the TNNP (132.5 m^2^ and 190.8 m^2^). These patches were measured using georeferencing and polygons were subsequently reconstructed in ArGIS.

### Fish assemblages

Considering the two periods studied, a total of 113 fish species belonging to 34 families were recorded in elkhorn coral formations, with Labridae, Haemulidae, Pomacentridae, and Serranidae as the most represented (Table 1). *Acanthurus coeruleus, A. tractus, Chaetodon ocellatus, Haemulon flavolineatum, Scarus iseri, Sparisoma viride, Thalassoma bifasciatum, Abudefduf saxatilis, Microspathodon chrysurus, Stegastes adustus*, and *Canthigaster rostrata* were common to all localities. These species had an observation frequency above 50%, with the exception of *A. tractus, C. ocellatus, S. iseri,* and *C. rostrata* (Table S2).

**Table 1:** Reef fish families recorded along the Colombian Caribbean, indicating the number of species per family found in each locality (2011–2012 and 2023–2024). Localities: Tayrona National Natural Park (TNNP), Isla Arena (IA), Rosario Islands (RI), San Bernardo Islands (SBI), Urabá Gulf (UG), Isla Fuerte (IF), and San Andrés Islands (SAI).

**Family**	**Total species count in 7 locations**	***A. palmata***							**Total species count in 6 locations**	***A. cervicornis***					
		**TNNP**	**IA**	**RI**	**SBI**	**UG**	**IF**	**SAI**	**TNNP**	**RI**	**SBI**	**UG**	**IF**	**SAI**
Acanthuridae	3	3	3	3	3	3	3	2	3	3	0	2	2	1	0
Aulostomidae	1	1		1	1	1	1	1	1	1		1	1		
Apogonidae	1							1							
Balistidae	1			1											
Blenniidae	2	1	1		1			2							
Carangidae	4	3	2	1	3	0	1	1	3	2		2	1	0	0
Chaenopsidae	1					1									
Chaetodontidae	4	4	2	2	3	3	3	3	3	3		2			
Cirrhitidae	1	1				1	1		1					1	
Diodontidae	2	2	1		1		1	1	2	2		1			
Ephippidae	1		1												
Gerreidae	1		1		1										
Gobiidae	3	3	1		1				1	1	1	1	1		
Ginglymostomidae									1			1			
Haemulidae	13	11	7	1	7	4	7	3	6	6	1	3	2	1	1
Holocentridae	4	4	0	2	3	3	2	2	3	3	0	2	1	1	1
Kyphosidae	1	1	1	1	1		1	1							
Labridae	18	16	8	8	14	13	16	9	15	10	1	9	10	6	2
Labrisomidae	2		1		1			1							
Lutjanidae	4	4	3	1	1	0	2	0	3	3	0	2	0	0	0
Monacanthidae	3	2		2	1		1		1			1			
Mugilidae	1	1													
Mullidae	2	2			1		1	1	2	2		1			
Muraenidae	2	2						1	1	1					
Ophichthidae									1	1					
Ostraciidae	4	4	0	1	1	0	1	0	2	1	0	1	0	0	0
Pempheridae	1	1	1			1	1								
Pomacanthidae	4	4	0	0	2	1	3	1	4	1	0	4	0	0	0
Pomacentridae	10	7	4	7	6	6	6	6	10	7	2	6	6	2	3
Priacanthidae	2	2			1		1		1			1		1	
Sciaenidae	2	1	1		1	1	2		2			2	2		
Scorpaenidae	2		1						1	1					
Serranidae	9	5	1	2	6	4	2	1	7	4	2	6	4	3	2
Sphyraenidae	1	1	1												
Synodontidae	1	1					1		2	2		1			
Tetraodontidae	2	1	1	1	1	1	1	1	2	2	1	1	1		1
**Total**	**113**	**88**	**42**	**34**	**61**	**43**	**58**	**38**	**78**	**47**	**5**	**40**	**24**	**13**	**7**

In staghorn coral patches, a total of 78 fish species were recorded, belonging to 25 families, with Labridae, Pomacentridae, Serranidae, and Haemulidae as the most abundant families (Table 1). The species common to all localities were *Stegastes planifrons, Cephalopholis cruentata*, and *Hypoplectrus puella. Thalassoma bifasciatum* was seen in all localities but San Andrés. Of the censuses conducted in the six localities, the only species whose frequency exceeded 75% was the threespot damselfish, *S. planifrons*; *H. flavolineatum, S. iseri*, and *Sparisoma aurofrenatum* were detected in at least three of the six localities (Table S3).

The highest fish richness in *A. palmata* patches was recorded in the TNNP (85 species), followed by Isla Fuerte (59) and San Bernardo Islands (55); Urabá Gulf (41) and San Andrés Islands, Isla Arena and Rosario Islands were the locations with the lowest number of species (fewer than 40). For the five locations with records in both sampling periods (2011–2012 and 2023–2024), a greater number of species was generally observed in the latter period (Fig. 3A). It is noteworthy that for Rosario Islands *T. bifasciatum* and *M. chrysurus* were the only species consistently recorded in the evaluated colonies. Although Isla Arena hosts many of the frequently observed species, they were never abundant, while *Chaetodon capistratus* and *Aulostomus maculatus*, common in all other localities, were absent. It should be mentioned that *Pomacanthus arcuatus, Diodon hystrix, Cantherhines pullus, Lutjanus mahogoni,* and *Haemulon plumierii* were absent from the Urabá Gulf. In San Andrés Island, *Acanthurus chirurgus, Bodianus rufus, Sparisoma rubripinne*, and *C. cruentata*, common in all other localities, were not recorded. Overall, the main trophic guilds, which included 73 species (67% of the total fish fauna), were invertivores (im, mobile 32.7%; is, sessile 11.5%), omnivores (om) (11.5%), and planktivores (11.5%). 84% of these fishes were benthopelagic, 13.4% were benthic, and 2.6% were pelagic.

**Figure 3:**
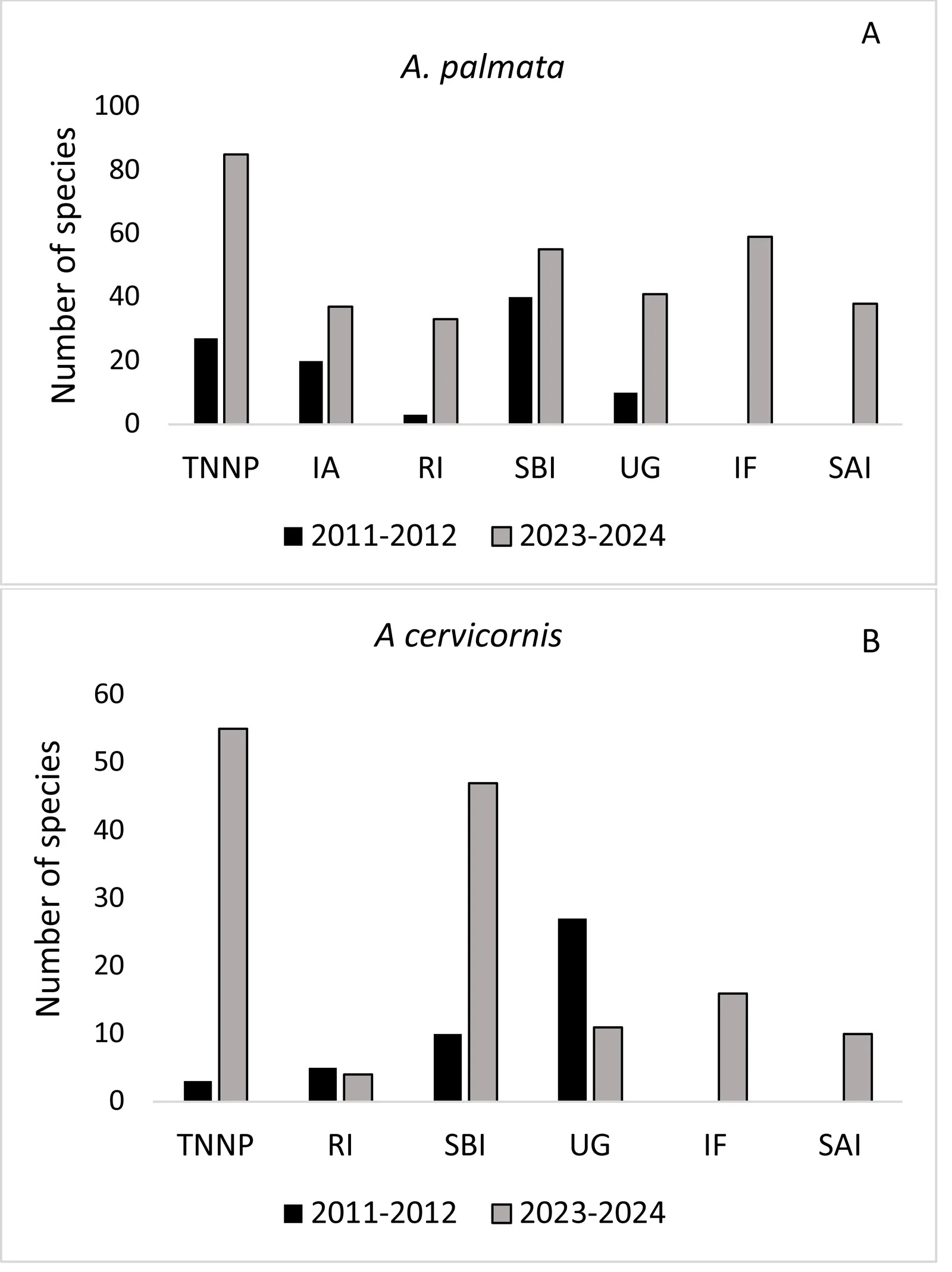
(A–B) Reef fish species richness between sites during the two sampling periods (2011–2012 and 2023–2024). TNNP Tayrona National Natural Park, RI Rosario Islands, SBI San Bernardo Islands, UG Urabá Gulf, IF Isla Fuerte, SAI San Andrés Islands.

The highest fish richness in *A. cervicornis* was recorded in the TNNP (55), followed by San Bernardo Islands (47). Isla Fuerte, Urabá Gulf, and San Andrés Islands had 16, 11 and 10 species, respectively, and Rosario Islands had the lowest number of species (4). It is worth highlighting the number of species for the Rosario Islands and the Gulf of Urabá locations between the two periods studied. In the former, the few scattered colonies maintained between four and five species, but in the latter, a clear reduction in the number of species was evident, as a result of colony mortality with the consequent patch loss (Fig. 3B). Some species generally common across localities were absent from specific sites: *H. flavolineatum* from Isla Fuerte, *T. bifasciatum* from San Andrés Islands, and *Holocentrus rufus*, *S. iseri* and *S. aurofrenatum* from Rosario Islands. The main guilds were invertivores (im, mobile 30.7%, and is, sessile 14.1%), omnivores (om) (11.5%), and planktivores (8.9%), including 51 species (65.3% of the total fish fauna). 85.9% of those fishes were benthopelagic, 11.5% were benthic, and 2.6% pelagic.

The *A. palmata* ANOVA showed differences between localities (*F*_6,37_ = 3.44; *p* = 0.0084), with the Urabá Gulf being distinct from the TNNP, Isla Fuerte, and San Bernardo Islands (Fig. 4A); no significant differences were found in *A. cervicornis* between localities (*F*_5,22_ = 2.34; *p* = 0.086). In relation to patch size, no significant differences were found in *A. palmata* (*p* = 0.14) (Fig. 4B); nevertheless, the analysis showed statistical differences in *A. cervicornis* (*F*_2,22_ = 13.08; *p* < 0.05) with moderate and large colonies differing from scattered ones.

**Figure 4:**
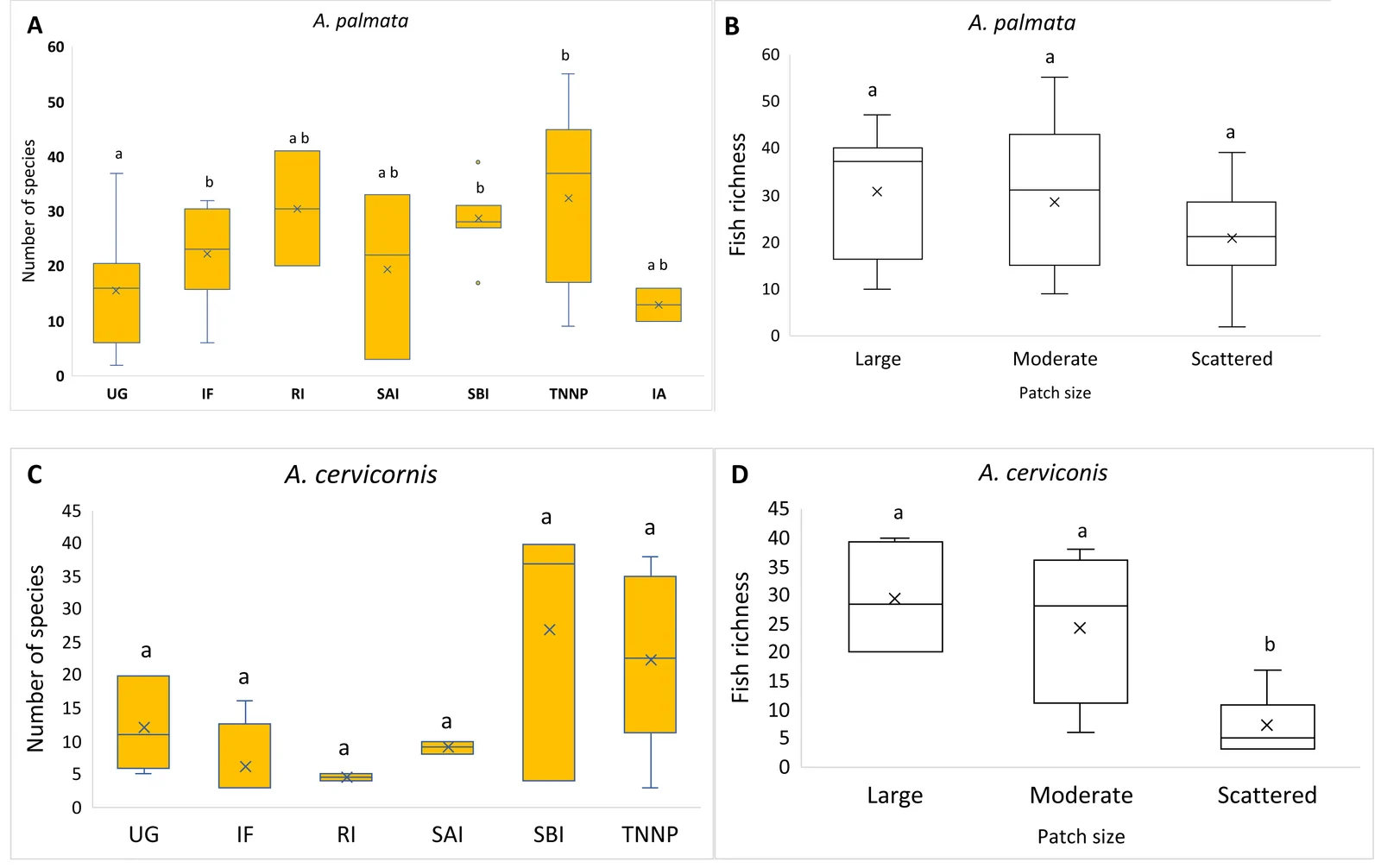
(A–C). Variation in the number of fish species across the study areas of the Colombian Caribbean (2011–2012 and 2023–2024). (B–D) Relationship between fish species richness and reef patch size (scattered, moderate, large) in *Acropora* species. TNNP Tayrona National Natural Park, RI Rosario Islands, SBI San Bernardo Islands, UG Urabá Gulf, IF Isla Fuerte, SAI San Andrés Islands.

Fish composition differed significantly between patch sizes in elkhorn coral (PERMANOVA, *p* = 0.016). However, dissimilarity values were similar across all comparisons (59.3% isolated and moderate, 63.4% isolated and large, 57.3% moderate and large), indicating the absence of marked contrasts between pairs of categories. Pairwise SIMPER analysis showed that no species contributed more than 3% to the dissimilarity between patch types. However, when examining the average contribution within each group, some species were more abundant in different patch sizes (eight species contributing more than 5% in small patches, eight in moderate patches, and seven in large patches), with *M. chrysurus*, *H. flavolineatum*, *A. saxatilis*, *S. adustus*, and *S. viride* being common to all three groups (Table S4).

In staghorn coral the pattern was similar, with significant differences in fish composition related to patch size (PERMANOVA, *p* = 0.005). However, dissimilarity in fish composition was high between small and moderate patches (74.1%) and between small and large patches (74.7%), while it was lower between moderate and large patches (54.2%). This indicates that small patches harbor markedly impoverished fish assemblages, while moderate and large patches exhibit more similar compositions. Furthermore, the observed dissimilarity (SIMPER analysis) was not driven by particular species, but rather by the cumulative contributions of multiple species with low individual contributions (less than 4.4%). The average contribution within each group showed that four species contributed more than 5% in small patches, eight in moderate patches, and 13 in large patches, but only *S. viride* and *S. planifrons* were common to all (Table S4).

## Discussion

The living status of *Acropora* colonies is critical throughout the Caribbean; nevertheless, nowadays the associated fish fauna seems to be resisting coral degradation in several localities. For *A. palmata*, the three-dimensional structure has been maintained, allowing most of the common fish species to be present in the studied formations. The bleaching event of 2023–2024 showed that although 100% of the colonies died in some areas, the composition of the associated fish showed no impact, suggesting that these reef areas should be reassessed to record the effect and identify the degree of impact. However, the *A. cervicornis* formations show a clear reduction in populations, and the loss of structure is also evident, indicating a decline in the associated reef fish assemblages.

Although multiple disturbances have occurred, altering coral structure, there are local differences in the pattern of decline of the two *Acropora* species. It is important to highlight that these assessments coincided with the widespread bleaching event of 2023 and 2024 (Cheng et al., 2024; Reimer et al., 2024; Doherty, Johnson & Goodbody-Gringley, 2025; Lopez et al., 2025), which resulted in mortality for *A. palmata* in areas such as the San Bernardo Islands and Isla Arena.

The greatest historical loss of both species in the Rosario Islands dates back to the late 20th century, with structural modifications caused by the input of sediment and nutrients from the Dique Channel (Gómez-Giraldo et al., 2009), which led to their near disappearance. In the San Bernardo Islands, the effects of the 2023 bleaching event resulted in 100% mortality of the georeferenced *A. palmata* colonies, while *A. cervicornis* has practically disappeared from the areas near the islands. However, in San Bernardo Islands significant live populations of this species are found far from the islets. Isla Arena is an exceptional location because it is covered most of the year by turbid waters from the Magdalena, the largest river discharging into the Caribbean Sea and the northern winds that typically begin in November clear the water (Pinzón, Perdomo & Díaz, 1998); nevertheless, the occurrence of high temperatures in 2023 produced the bleaching of the entire formation, with a mortality rate close to 50%, and in 2024 bleaching occurred again reaching 100% of the colonies. The current status of this unique formation is still unknown. Staghorn coral has not been found alive since 2011–2012 in that locality. In the Urabá Gulf, *A. palmata*, although found only in scattered patches, is significantly abundant in all reef areas; the loss of large patches of *A. cervicornis,* such as those of El Aguacate and La Mora, is evident compared to what was recorded by García-Urueña & Garzón-Machado (2020), but the entire formation bleached during the 2023 temperature increase, with high mortality.

The mortality of both *Acropora* species in the oceanic San Andrés archipelago is evident, including the complete disappearance of the large patch of *A. cervicornis* in Cayo Bolívar, leaving only scattered, shallow colonies near the main island. San Andrés is the only Colombian locality whose reefs have shown coral mortality due to tissue loss disease (Aeby et al., 2024); however, the reduction began well before the discovery of the disease. This contrasts sharply with the vigorous elkhorn coral formations in the TNNP, where seasonal upwelling occurs, lowering sea surface temperatures to at least 22 °C (Alvarado-Jiménez, Rincón-Díaz & García-Urueña, 2024), providing a refuge for this species. Staghorn coral, while subject to the same climatic regime, faces threats more related to storm surges and human impacts, such as the use of fishing gear that compromises existing patches (García-Urueña, Garzón-Machado & Zárate-Arévalo, 2025). Nevertheless, small patches resulting from asexual restoration programs of these species have increased the species’ coverage (Sánchez, Garzón-Machado & García-Urueña, 2025).

Climate change-induced coral bleaching has caused massive devastation to coral reef habitats (Hoegh-Guldberg, 1999), and its frequency and severity were predicted to increase in recent decades (Donner et al., 2005; Hughes et al., 2018; Dietzel et al., 2021). However, in 2023 and 2024, unprecedented water temperatures and cumulative heat stress precipitated coral bleaching levels approaching 100%, a phenomenon that occurred across more than one fifth of the world’s land area (Cheng et al., 2024; Reimer et al., 2024; Doherty, Johnson & Goodbody-Gringley, 2025; Lopez et al., 2025; Manzello et al., 2025). Our observations on live colonies, made before, during, and after the bleaching event, indicate that the strong relationship between *Acropora* structures and fish assemblages is maintained even after coral mortality due to bleaching. The coral formations in San Bernardo islands may be a good example of this, since they maintain the second richest fish fauna of the studied localities, despite the death of 100% of the colonies of *A. palmata* in 2023. In any case, the loss of the structures, is likely to have severe consequence for the diversity and abundance of fish assemblages living in the continental and insular shelves.

Unlike bleaching, the greatest impact on associated fish communities has been shown to occur when colonies are destroyed or removed, reducing topography and resulting in a loss of structure. This indicates that many of the long-term effects of coral mortality on reef fish communities will be delayed until the physical tridimensional structure provided by corals is lost (Adam et al., 2014; Gavriel et al., 2023; Lalas et al., 2024). According to García-Urueña & Garzón-Machado (2020), between 2016 and 2018, *A. cervicornis* populations in the Urabá Gulf were the most important in the Colombian continental Caribbean, but surveys between 2023 and 2024 showed a clear loss of structure and mortality, highlighting its impact on fish communities in less than a decade, as evidenced by a reduction in fish richness.

Considering that the number of fish species reported from the Colombian Caribbean has been estimated at 1,100–1,200 (Acero & Polanco, 2017), we recorded approximately 10% of this richness living in the studied *Acropora* patches. It should be emphasized that those formations cover far less than 0.1% of the continental and insular Colombian shelves (Díaz et al., 2000). Several authors have gathered information supporting the importance of living or at least standing coral structures for healthy reef fish communities, including commercial species (Coker, Graham & Pratchett, 2012; Emslie, Cheal & Johns, 2014; Rogers et al., 2018). Our findings support these claims for *A. cervicornis*, particularly in the Gulf of Urabá, where the loss of the structure is linked to the disappearance of fish communities. Lirman (1999) emphasized the relevance of species such as *M. chrysurus*, *Stegastes partitus, S. adustus* (as *S. fuscus*), and *S. planifrons*, as well as schools of grunts and snappers that utilize standing structures of *A. palmata*. Lemoine & Valentine (2012) examined in detail the populations of the threespot damselfish and their influence on the main herbivore groups (surgeonfishes and parrotfishes); this pomacentrid is usually very abundant in Colombian elkhorn coral, except in Rosario Islands.

The analysis showed that no single fish species contributed more than 5% to elkhorn coral patch size dissimilarity, indicating that patch differences are distributed among many species with small individual contributions. Of the 11 common fish species, *M. chrysurus*, *H. flavolineatum*, *S. adustus*, *A. saxatilis*, and *S. viride* showed the greatest contribution in the three types of patches evaluated, reflecting abundance patterns characteristic of each patch group, without any marked differences between them. Graham & Nash (2013) found that Pomacanthidae, Pomacentridae, and Scaridae presented a positive relationship with increasing reef structural complexity; our data indicate that the number of pomacentrid and labrid species remain unchanged regardless of *Acropora* patch sizes.

On the other hand, due to the severe degradation of staghorn coral, fish assemblages from small patches are clearly separated from those occurring in moderate and large patches; moderate and large patches show greater similarity, suggesting a threshold effect in habitat size structuring fish communities. This demonstrates patch reduction, in terms of richness and structure, harboring poorer reef fish assemblages. Furthermore, the observed dissimilarity was not dominated by particular species, but rather by the cumulative contribution of multiple species with low individual contributions. Taken together, these results suggest that the effect of patch size on fish communities is significant but diffuse, with high overlap in composition between categories. The analysis showed that three fish species are the most frequently observed in most patches (*S. planifrons, C. cruentata*, and *H. puella*); nevertheless, only the threespot damselfish made a significant contribution in all three types of patches, while *C.* c*ruentata* and *T. bifasciatum* contributed to the large and moderate patches. These results show that the main characteristic of the reduction of fish assemblages is the loss of the structure of these branched species, agreeing with numerous studies (Chong-Seng et al., 2012; Arias-Godínez et al., 2021; Lalas et al., 2024).

Variations of the fish assemblages dwelling in *Acropora* patches along the Colombian continental Caribbean coast is difficult to detect due to the general mortality of reefs in recent years. TNNP elkhorn coral formations, the best conserved in the country, hold unsurprisingly the richest fish assemblages, but the most ubiquitous and abundant species are noncommercial fishes (*A. maculatus, Brachygenys chrysargyrea, S. aurofrenatum, S. iseri, S. planifrons, Azurina cyanea, Mulloidichthys martinicus*, and *Myripristis jacobus*), which may be an indicator of the overexploitation on the TNNP reefs.

Many fish species were detected occurring in only one locality. However, in most cases it is clear that the situation is not related to some habitat requirement, but it likely reflects a random circumstance. Nevertheless, some of the cases stand comments. The few surviving colonies of *A. palmata* in Rosario Islands were the only sites where *Melichthys niger* was seen. That species, typical of oceanic or very clear water reefs (Robertson & Van Tassell, 2023), is also abundant in San Andrés; however, the only small patches of elkhorn coral patch surviving in San Andres are located in very shallow waters (<2 m) and close to shore (<10 m), where that species is not typically found. Elkhorn coral formations in Isla Arena lived in extreme conditions in a murky continental environment (Sierra-Escrigas, Peluffo & García-Urueña, 2020). Hence it is not unusual that fish species with continental affinities such as *Chaetodipterus faber* and *Paranisotremus moricandi* were found only there. Isla Arena hosted most of the frequently observed species, which were never abundant; while *C. capistratus* and *T. bifasciatum* were absent. That chaetodontid has been postulated as an indicator of reef health due to its coral polyp feeding habits (Acero & Rivera, 1992).

The TNNP, on the other hand, is a somewhat paradoxical locality. It is clearly continental, as shown by the presence of fish species with those affinities, such as *Strongylura notata*, *Mugil curema*, and *Sphoeroides spengleri*, but at the same time contains the best-preserved coral formations in the continental coast of Colombia (García-Urueña, Garzón-Machado & Sierra-Escrigas, 2020). The importance of those formations is remarked by the occurrence of some carnivorous fish species only detected in the TNNP, such as *Gymnothorax moringa* (in both *Acropora* species), *Ophichthus ophis*, and *Cephalopholis fulva* (both in staghorn coral), and *Mycteroperca acutirostris* and *M. bonaci* (both in elkhorn coral).

Regardless of the destruction of *A. palmata* formations in San Bernardo Islands, they still maintained an important reef fish fauna. Species worth mentioning are two *Hypoplectrus* (*H. affinis* in elkhorn coral and *H. randallorum* in staghorn coral), and *Ginglymostoma cirratum* and *Acanthostracion quadricornis*, both in staghorn coral. The status of *A. palmata* in the Urabá Gulf is the most critical, showing marked differences compared to the reefs of Isla Fuerte, TNNP, and the San Bernardo Islands. Urabá reefs have already been characterized with a richness of 212 fish species (Escobar-Sierra et al., 2021); however, our records showed only 44 species in *A. palmata* and 11 in *A. cervicornis*, with absence of species that were common in the different environments. The lack of records of *L. mahogoni* and *H. plumierii*, two commercially important fish species typical of shallow reefs (Robertson & Van Tassell, 2023), from the patches visited by us may be related to their degradation. Furthermore, Urabá Gulf is the only place where the reduction in number of fish species in the staghorn coral was evident.

Most fishes inhabiting *Acropora* reefs are benthopelagic species, usually highly mobile, but with relatively restricted home areas. Those species eat mainly mobile invertebrates, but other food sources such as fishes, sessile invertebrates, and plant material are also important (Randall, 1967; Emslie, Cheal & Johns, 2014). Most of those fishes seem to be able to survive even if corals are completely dead, while the reef structure is still erected. However, once the reef is totally destroyed, many species that depend on the refuge provided by the reef and/or the food items dwelling around the structures would likely experience significant reduction in their populations (Coker, Graham & Pratchett, 2012; Emslie, Cheal & Johns, 2014).

Our research emphasizes the immense value of *Acropora* formations for the maintenance of fish biodiversity. Key ecological groups, such as wrasses (including parrotfishes), surgeonfishes, and damselfishes depend strongly on healthy, or at least standing, coral structures. Fishes of great economic importance, such as snappers, groupers, and grunts use *Acropora* colonies for refuge at least during a part of their lives (Robertson & Van Tassell, 2023). Coastal human populations depend on the coral-fish relationship for food security as well as for tourism initiatives which may bring additional income to their activities. Authorities and civil organizations should increase the level of protection on coral reefs in both Colombian coasts before they disappear entirely.

## Conclusions

*Acropora palmata* and *A. cervicornis* populations in the Colombian Caribbean show a general reduction in their populations; however, there are local differences for the two species, which coincided with the widespread bleaching event of 2023 and 2024. For *A. palmata*, the reef was devastated in some locations, but the composition of associated fish showed no impact, suggesting that these reef areas need to be reassessed to record the effect and identify the degree of impact. For *A. cervicornis*, on the other hand, the mortality is evident, as is the loss of its associated reef fish community. A total of 122 fish species were found associated with *Acropora*. It may be worth emphasizing the lack or scarcity of medium and large sized species, which are fishermen’s targets. While the coral three dimensions structure is standing, most frequent fish species were present in the surveyed formations; however, *A. cervicornis* patches tend to disappear faster than *A. palmata* ones, causing fish assemblages to be clearly reduced.

## Supplemental Information

10.7717/peerj.21744/supp-1 Supplemental Information 1 Sampled locations and reefs along the Colombian Caribbean (2011–2012 and 2023–2024) TNNP Tayrona National Natural Park, IR Rosario Islands, IA Isla Arena, SB San Bernardo Islands, IF Isla Fuerte, GU Urabá Gulf.

10.7717/peerj.21744/supp-2 Supplemental Information 2 Frequency of observation of reef fish associated with *Acropora palmata* along the Colombian Caribbean (2011–2012 and 2023–2024) Position in water column: benthic (bottom), benthopelagic (low), pelagic (high). Diet: herbivore-detritivore (hd), macroalgal feeder (hm), sessile invertebrates (is), movil invertebrates (im), planktonic (pk), omnivore (om), and piscivore (fc).

10.7717/peerj.21744/supp-3 Supplemental Information 3 Frequency of observation of reef fish associated with *Acropora cervicornis* along the Colombian Caribbean (2011–2012 and 2023–2024) Position in water column: benthic (bottom), benthopelagic (low), pelagic (high). Diet: herbivore-detritivore (hd), macroalgal feeder (hm), sessile invertebrates (is), movil invertebrates (im), planktonic (pk), omnivore (om), and piscivore (fc).

10.7717/peerj.21744/supp-4 Supplemental Information 4 Percentage contribution values of fish species included until a cumulative contribution of 70% Values from the similarity percentages (SIMPER) analysis showing the mean similarity within patch size and the percentage contribution of fish species.

## Additional Information and Declarations

### Field Study Permissions

The following information was supplied relating to field study approvals (i.e., approving body and any reference numbers): Scientific research and biological diversity permit PIC 004 in September 2021.

### Data Availability

The following information was supplied regarding data availability: The data is available in the Supplemental Files.
