# Supplementary material for: Reef fish assemblages on Acropora patches in the Colombian Caribbean: resistance to reef degradation
Source: PeerJ. 2026 Oct 5;14:e21744. doi: 10.7717/peerj.21744 (PMC13648214; doi:10.7717/peerj.21744)
Supplement: Supplementary file 1 [file peerj-14-21744-s002.docx]

| **Species** | **UG** | **IA** | **IF** | **RI** | **SAI** | **SBI** | **TNNP** | **Water column position** | **Diet** |
| --- | --- | --- | --- | --- | --- | --- | --- | --- | --- |
| Acanthurus chirurgus | 11,1 | 50,0 | 33,3 | 50,0 | 0,0 | 50,0 | 28,5 | low | hd |
| Acanthurus coeruleus | 66,7 | 50,0 | 83,3 | 50,0 | 100,0 | 87,5 | 78,6 | low | hm |
| Acanthurus tractus | 44,4 | 50,0 | 66,7 | 50,0 | 100,0 | 50,0 | 57,1 | low | hd |
| Aulostomus maculatus | 22,2 | 0,0 | 66,7 | 50,0 | 50,0 | 50,0 | 78,6 | low | fc |
| Apogon maculatus | 0,0 | 0,0 | 0,0 | 0,0 | 50,0 | 0,0 | 0,0 | low | pk |
| Melichthys niger | 0,0 | 0,0 | 0,0 | 50,0 | 0,0 | 0,0 | 0,0 | low, high | om |
| Ophioblennius macclurei | 0,0 | 50,0 | 0,0 | 0,0 | 50,0 | 87,5 | 50,0 | bottom | om |
| Entomacrodus nigricans | 0,0 | 0,0 | 0,0 | 0,0 | 50,0 | 0,0 | 0,0 | bottom | om |
| Caranx bartholomei | 0,0 | 0,0 | 0,0 | 0,0 | 0,0 | 12,5 | 0,0 | low, high | fc |
| Caranx crysos | 0,0 | 50,0 | 0,0 | 0,0 | 0,0 | 12,5 | 28,5 | high | fc |
| Caranx ruber | 0,0 | 100,0 | 16,7 | 50,0 | 50,0 | 37,5 | 57,1 | low, high | fc |
| Decapterus sp. | 0,0 | 0,0 | 0,0 | 0,0 | 0,0 | 0,0 | 7,1 | high | pk |
| Acanthemblemaria rivasi | 11,1 | 0,0 | 0,0 | 0,0 | 0,0 | 0,0 | 7,1 | bottom | pk |
| Chaetodon capistratus | 44,4 | 0,0 | 100,0 | 50,0 | 100,0 | 75,0 | 50,0 | low | is |
| Chaetodon ocellatus | 11,1 | 50,0 | 33,3 | 50,0 | 50,0 | 62,5 | 35,7 | low | is |
| Chaetodon striatus | 11,1 | 50,0 | 16,7 | 0,0 | 100,0 | 12,5 | 14,2 | low | is |
| Chaetodon sedentarius | 0,0 | 0,0 | 0,0 | 0,0 | 0,0 | 0,0 | 57,1 | low | is |
| Amblycirrhitus pinos | 11,1 | 0,0 | 16,7 | 0,0 | 0,0 | 0,0 | 14,2 | bottom | im |
| Diodon holocanthus | 0,0 | 0,0 | 0,0 | 0,0 | 0,0 | 25,0 | 28,6 | low | is |
| Diodon hystrix | 0,0 | 50,0 | 16,7 | 0,0 | 50,0 | 0,0 | 21,4 | low | is |
| Chaetodiptetus faber | 0,0 | 50,0 | 0,0 | 0,0 | 0,0 | 0,0 | 0,0 | low, high | om |
| Gerres cinereus | 0,0 | 50,0 | 0,0 | 0,0 | 0,0 | 12,5 | 0,0 | low | im |
| Coryphopterus personatus | 0,0 | 0,0 | 0,0 | 0,0 | 0,0 | 12,5 | 14,2 | bottom | pk |
| Elacatinus illecebrosus | 0,0 | 50,0 | 0,0 | 0,0 | 0,0 | 0,0 | 28,5 | bottom | limpiador |
| Gnatholepis thompsoni | 0,0 | 0,0 | 0,0 | 0,0 | 0,0 | 0,0 | 7,1 | bottom | im |
| Anisotremus surinamensis | 0,0 | 50,0 | 0,0 | 0,0 | 0,0 | 0,0 | 21,4 | low | im |
| Paranisotremus moricandi | 0,0 | 50,0 | 0,0 | 0,0 | 0,0 | 0,0 | 0,0 | low | im |
| Anisotremus virginicus | 22,2 | 50,0 | 33,3 | 0,0 | 0,0 | 0,0 | 7,1 | low | im |
| Haemulon aurolineatum | 11,1 | 0,0 | 33,3 | 0,0 | 0,0 | 12,5 | 28,5 | low | im |
| Haemulon bonariensis | 0,0 | 50,0 | 0,0 | 0,0 | 0,0 | 0,0 | 0,0 | low | im |
| Haemulon carbonarium | 22,2 | 100,0 | 66,7 | 0,0 | 50,0 | 62,5 | 42,8 | low | im |
| Haemulon flavolineatum | 55,6 | 100,0 | 100,0 | 50,0 | 100,0 | 100,0 | 100,0 | low | im |
| Haemulon macrostomum | 0,0 | 100,0 | 33,3 | 0,0 | 0,0 | 75,0 | 42,8 | low | im |
| Haemulon melanurum | 0,0 | 0,0 | 0,0 | 0,0 | 0,0 | 0,0 | 7,1 | low | im |
| Haemulon plumierii | 0,0 | 0,0 | 0,0 | 0,0 | 0,0 | 50,0 | 21,4 | low | im |
| Haemulon sciurus | 0,0 | 0,0 | 16,7 | 0,0 | 0,0 | 25,0 | 7,1 | low | im |
| Brachygenys chrysargyrea | 0,0 | 0,0 | 33,3 | 0,0 | 50,0 | 75,0 | 85,7 | low | im |
| Haemulon striatum | 0,0 | 0,0 | 0,0 | 0,0 | 0,0 | 0,0 | 7,1 | low, high | pk |
| Holocentrus adscensionis | 22,2 | 0,0 | 0,0 | 50,0 | 50,0 | 75,0 | 28,5 | low | im |
| Holocentrus rufus | 0,0 | 0,0 | 83,3 | 0,0 | 0,0 | 37,5 | 57,1 | low | im |
| Myripristis jacobus | 44,4 | 0,0 | 33,3 | 50,0 | 0,0 | 37,5 | 64,2 | low, high | pk |
| Neoniphon vexillarium | 22,2 | 0,0 | 0,0 | 0,0 | 100,0 | 0,0 | 7,1 | low | im |
| Kyphosus sectatrix | 0,0 | 100,0 | 16,7 | 50,0 | 50,0 | 87,5 | 21,4 | low, high | hm |
| Bodianus parrae | 11,1 | 0,0 | 33,3 | 0,0 | 0,0 | 12,5 | 14,2 | high | pk |
| Bodianus rufus | 11,1 | 50,0 | 50,0 | 50,0 | 0,0 | 12,5 | 85,7 | low | im |
| Halichoeres bivittatus | 11,1 | 100,0 | 50,0 | 0,0 | 50,0 | 75,0 | 7,1 | low | im |
| Halichoeres garnoti | 11,1 | 0,0 | 50,0 | 50,0 | 0,0 | 25,0 | 21,4 | low | im |
| Halichoeres maculipinna | 11,1 | 0,0 | 33,3 | 0,0 | 50,0 | 12,5 | 21,4 | low | im |
| Halichoeres radiatus | 0,0 | 50,0 | 50,0 | 0,0 | 50,0 | 37,5 | 14,2 | low | im |
| Scarus coelestinus | 0,0 | 0,0 | 0,0 | 0,0 | 0,0 | 0,0 | 7,1 | low | hm |
| Scarus guacamaia | 0,0 | 0,0 | 16,7 | 0,0 | 0,0 | 25,0 | 57,1 | low | hm |
| Scarus iseri | 44,4 | 50,0 | 100,0 | 50,0 | 50,0 | 100,0 | 64,2 | low | hm |
| Scarus taeniopterus | 0,0 | 0,0 | 16,7 | 50,0 | 0,0 | 25,0 | 21,4 | low | hm |
| Scarus vetula | 11,1 | 0,0 | 16,7 | 50,0 | 50,0 | 0,0 | 50,0 | low | hm |
| Sparisoma atomarium | 0,0 | 0,0 | 0,0 | 0,0 | 50,0 | 37,5 | 0,0 | low | hd |
| Sparisoma aurofrenatum | 22,2 | 0,0 | 50,0 | 0,0 | 50,0 | 62,5 | 64,2 | low | hd |
| Sparisoma chrysopterum | 22,2 | 100,0 | 16,7 | 0,0 | 0,0 | 37,5 | 28,5 | low | hd |
| Sparisoma rubripinne | 11,1 | 100,0 | 66,7 | 50,0 | 0,0 | 62,5 | 57,1 | low | hd |
| Sparisoma viride | 66,7 | 100,0 | 100,0 | 50,0 | 100,0 | 87,5 | 92,8 | low | hm |
| Thalassoma bifasciatum | 66,7 | 50,0 | 83,3 | 100,0 | 100,0 | 100,0 | 57,1 | low | im |
| Halichoeres poeyi | 22,2 | 0,0 | 16,7 | 0,0 | 0,0 | 0,0 | 0,0 | low | im |
| Labrisomus nuchipinnis | 0,0 | 100,0 | 0,0 | 0,0 | 0,0 | 0,0 | 0,0 | bottom | im |
| Malacoctenus triangulatus | 0,0 | 0,0 | 0,0 | 0,0 | 100,0 | 12,5 | 0,0 | bottom | im |
| Lutjanus apodus | 0,0 | 100,0 | 50,0 | 0,0 | 0,0 | 0,0 | 50,0 | low | im |
| Lutjanus griseus | 0,0 | 50,0 | 0,0 | 0,0 | 0,0 | 0,0 | 14,2 | low | im |
| Lutjanus jocu | 0,0 | 0,0 | 0,0 | 0,0 | 0,0 | 12,5 | 28,5 | low | fc |
| Lutjanus mahogoni | 0,0 | 50,0 | 83,3 | 50,0 | 0,0 | 0,0 | 42,8 | low | im |
| Ocyurus chrysurus | 0,0 | 0,0 | 33,3 | 0,0 | 0,0 | 0,0 | 0,0 | high | im |
| Cantherhines macrocerus | 0,0 | 0,0 | 0,0 | 50,0 | 0,0 | 0,0 | 0,0 | low | om |
| Cantherhines pullus | 0,0 | 0,0 | 16,7 | 50,0 | 0,0 | 25,0 | 42,8 | low | om |
| Aluterus scriptus | 0,0 | 0,0 | 0,0 | 0,0 | 0,0 | 0,0 | 7,1 | low, high | om |
| Mugil curema | 0,0 | 0,0 | 0,0 | 0,0 | 0,0 | 0,0 | 7,1 | low | detritivore |
| Mulloidichthys martinicus | 0,0 | 0,0 | 33,3 | 0,0 | 50,0 | 12,5 | 71,4 | low | im |
| Pseudupeneus maculatus | 0,0 | 0,0 | 0,0 | 0,0 | 0,0 | 0,0 | 21,4 | low | im |
| Gymnothorax miliaris | 0,0 | 0,0 | 0,0 | 0,0 | 100,0 | 0,0 | 7,1 | bottom | fc |
| Gymnothorax moringa | 0,0 | 0,0 | 0,0 | 0,0 | 0,0 | 0,0 | 7,1 | bottom | fc |
| Lactophrys bicaudalis | 0,0 | 0,0 | 0,0 | 0,0 | 0,0 | 25,0 | 14,2 | low | is |
| Lactophrys triqueter | 0,0 | 0,0 | 0,0 | 50,0 | 0,0 | 0,0 | 28,5 | low | is |
| Acanthostracion polygonium | 0,0 | 0,0 | 16,7 | 0,0 | 0,0 | 0,0 | 35,7 | low | is |
| Acanthostracion quadricornis | 0,0 | 0,0 | 0,0 | 0,0 | 0,0 | 0,0 | 7,1 | low | is |
| Pempheris schomburgkii | 11,1 | 50,0 | 16,7 | 0,0 | 0,0 | 0,0 | 57,1 | low | pk |
| Holacanthus ciliaris | 0,0 | 0,0 | 50,0 | 0,0 | 0,0 | 25,0 | 7,1 | low | is |
| Holacanthus tricolor | 0,0 | 0,0 | 16,7 | 0,0 | 0,0 | 0,0 | 21,4 | low | is |
| Pomacanthus arcuatus | 0,0 | 0,0 | 0,0 | 0,0 | 0,0 | 0,0 | 7,1 | low | is |
| Pomacanthus paru | 11,1 | 0,0 | 33,3 | 0,0 | 50,0 | 50,0 | 63,2 | low | is |
| Abudefduf saxatilis | 55,6 | 100,0 | 100,0 | 50,0 | 100,0 | 100,0 | 85,7 | low, high | pk |
| Azurina cyanea | 22,2 | 0,0 | 0,0 | 50,0 | 0,0 | 0,0 | 71,4 | low, high | pk |
| Azurina multilineata | 0,0 | 0,0 | 16,7 | 50,0 | 100,0 | 0,0 | 57,1 | low, high | pk |
| Microspathodon chrysurus | 77,8 | 100,0 | 100,0 | 100,0 | 100,0 | 100,0 | 100,0 | low | om |
| Stegastes adustus | 100,0 | 100,0 | 100,0 | 50,0 | 100,0 | 100,0 | 85,7 | low | om |
| Stegastes diencaeus | 0,0 | 0,0 | 0,0 | 50,0 | 0,0 | 25,0 | 0,0 | low | om |
| Stegastes partitus | 0,0 | 0,0 | 33,3 | 50,0 | 50,0 | 25,0 | 64,2 | low | om |
| Stegastes planifrons | 22,2 | 100,0 | 100,0 | 0,0 | 0,0 | 62,5 | 92,8 | low | om |
| Chromis insolata | 11,1 | 0,0 | 0,0 | 0,0 | 0,0 | 0,0 | 0,0 | low | pk |
| Abudefduf taurus | 0,0 | 0,0 | 0,0 | 0,0 | 50,0 | 0,0 | 0,0 | low | im |
| Heteropriacanthus cruentatus | 0,0 | 0,0 | 33,3 | 0,0 | 0,0 | 12,5 | 14,2 | low, high | pk |
| Priacanthus arenatus | 0,0 | 0,0 | 0,0 | 0,0 | 0,0 | 0,0 | 14,2 | low, high | pk |
| Odontoscion dentex | 11,1 | 50,0 | 66,7 | 0,0 | 0,0 | 25,0 | 21,4 | low | im |
| Eques punctatus | 0,0 | 0,0 | 33,3 | 0,0 | 0,0 | 0,0 | 0,0 | low | im |
| Scorpaena plumierii | 0,0 | 50,0 | 0,0 | 0,0 | 0,0 | 0,0 | 0,0 | bottom | fc |
| Pterois volitans | 22,2 | 0,0 | 16,7 | 0,0 | 0,0 | 0,0 | 0,0 | bottom | fc |
| Cephalopholis cruentata | 44,4 | 50,0 | 83,3 | 50,0 | 0,0 | 12,5 | 57,1 | low | im |
| Hypoplectrus affinis | 0,0 | 0,0 | 0,0 | 0,0 | 0,0 | 12,5 | 0,0 | low | im |
| Hypoplectrus nigricans | 11,1 | 0,0 | 0,0 | 0,0 | 0,0 | 75,0 | 0,0 | low | im |
| Hypoplectrus puella | 11,1 | 0,0 | 50,0 | 0,0 | 0,0 | 87,5 | 21,4 | low | im |
| Hypoplectrus unicolor | 0,0 | 0,0 | 0,0 | 0,0 | 0,0 | 12,5 | 7,1 | low | im |
| Mycteroperca acutirostris | 0,0 | 0,0 | 0,0 | 0,0 | 0,0 | 0,0 | 7,1 | low | fc, |
| Mycteroperca | 0,0 | 0,0 | 0,0 | 0,0 | 0,0 | 0,0 | 7,1 | low | fc |
| Serranus tigrinus | 11,1 | 0,0 | 0,0 | 50,0 | 0,0 | 25,0 | 0,0 | low | im |
| Rypticus saponaceus | 0,0 | 0,0 | 0,0 | 0,0 | 50,0 | 0,0 | 0,0 | low | im |
| Sphyraena barracuda | 0,0 | 50,0 | 0,0 | 0,0 | 0,0 | 0,0 | 21,4 | low, high | fc |
| Synodus intermedius | 0,0 | 0,0 | 16,7 | 0,0 | 0,0 | 0,0 | 28,5 | bottom | fc |
| Canthigaster rostrata | 11,1 | 50,0 | 66,7 | 50,0 | 100,0 | 62,5 | 64,2 | low | om |
