# Supplementary material for: Reef fish assemblages on Acropora patches in the Colombian Caribbean: resistance to reef degradation
Source: PeerJ. 2026 Oct 5;14:e21744. doi: 10.7717/peerj.21744 (PMC13648214; doi:10.7717/peerj.21744)
Supplement: Supplementary file 2 [file peerj-14-21744-s003.docx]

| **Species** | **UG** | **IF** | **RI** | **SAI** | **SBI** | **TNNP** | **Water column position** | **Diet** |
| --- | --- | --- | --- | --- | --- | --- | --- | --- |
| Acanthurus chirurgus | 0,0 | 0,0 | 0,0 | 0,0 | 0,0 | 50,0 | low | hd |
| Acanthurus coeruleus | 33,3 | 25,0 | 0,0 | 0,0 | 50,0 | 33,3 | low | hm |
| Acanthurus tractus | 33,3 | 0,0 | 0,0 | 0,0 | 50,0 | 50,0 | low | hd |
| Aulostomus maculatus | 33,3 | 0,0 | 0,0 | 0,0 | 75,0 | 66,6 | low | fc |
| Caranx bartholomei | 0,0 | 0,0 | 0,0 | 0,0 | 0,0 | 16,7 | low, high | fc |
| Caranx crysos | 0,0 | 0,0 | 0,0 | 0,0 | 25,0 | 0,0 | high | fc |
| Caranx ruber | 16,6 | 0,0 | 0,0 | 0,0 | 50,0 | 33,3 | low, high | fc |
| Chaetodon capistratus | 0,0 | 0,0 | 0,0 | 0,0 | 50,0 | 16,7 | low | is |
| Chaetodon ocellatus | 0,0 | 0,0 | 0,0 | 0,0 | 50,0 | 66,6 | low | is |
| Chaetodon striatus | 0,0 | 0,0 | 0,0 | 0,0 | 0,0 | 50,0 | low | is |
| Amblycirrhitus pinos | 0,0 | 0,0 | 0,0 | 0,0 | 0,0 | 0,0 | bottom | im |
| Diodon holocanthus | 0,0 | 0,0 | 0,0 | 0,0 | 25,0 | 16,6 | low | is |
| Diodon hystrix | 0,0 | 0,0 | 0,0 | 0,0 | 0,0 | 16,6 | low | is |
| Coryphopterus personatus | 16,6 | 0,0 | 50,0 | 0,0 | 50,0 | 16,6 | bottom | pk |
| Coryphopterus sp. | 0,0 | 0,0 | 0,0 | 0,0 | 25,0 | 0,0 | bottom | pk |
| Ginglymostoma cirratum | 0,0 | 0,0 | 0,0 | 0,0 | 25,0 | 0,0 | bottom, low | im |
| Haemulon aurolineatum | 0,0 | 0,0 | 0,0 | 0,0 | 0,0 | 16,6 | low | im |
| Haemulon carbonarium | 0,0 | 0,0 | 0,0 | 0,0 | 0,0 | 16,6 | low | im |
| Haemulon flavolineatum | 33,3 | 0,0 | 50,0 | 100,0 | 75,0 | 83,3 | low | im |
| Haemulon macrostomum | 16,6 | 0,0 | 0,0 | 0,0 | 0,0 | 0,0 | low | im |
| Haemulon plumierii | 0,0 | 25,0 | 0,0 | 0,0 | 50,0 | 83,3 | low | im |
| Brachygenys chrysargyreum | 0,0 | 0,0 | 0,0 | 0,0 | 25,0 | 50,0 | low | im |
| Holocentrus adscensionis | 0,0 | 0,0 | 0,0 | 0,0 | 25,0 | 16,6 | low | im |
| Holocentrus rufus | 16,6 | 50,0 | 0,0 | 100,0 | 50,0 | 33,3 | low | im |
| Myripristis jacobus | 0,0 | 0,0 | 0,0 | 0,0 | 0,0 | 33,3 | low, high | pk |
| Bodianus parrae | 16,6 | 25,0 | 0,0 | 0,0 | 0,0 | 16,7 | high | pk |
| Bodianus rufus | 33,3 | 0,0 | 0,0 | 0,0 | 25,0 | 66,6 | low | im |
| Halichoeres bivittatus | 0,0 | 0,0 | 0,0 | 0,0 | 50,0 | 50,0 | low | im |
| Halichoeres garnoti | 33,3 | 0,0 | 0,0 | 0,0 | 50,0 | 16,7 | low | im |
| Halichoeres maculipinna | 16,6 | 0,0 | 0,0 | 0,0 | 0,0 | 0,0 | low | im |
| Scarus iseri | 33,3 | 50,0 | 0,0 | 100,0 | 75,0 | 83,3 | low | hm |
| Scarus taeniopterus | 16,6 | 0,0 | 0,0 | 0,0 | 25,0 | 0,0 | low | hm |
| Sparisoma atomarium | 0,0 | 0,0 | 0,0 | 0,0 | 25,0 | 0,0 | low | hd |
| Sparisoma aurofrenatum | 33,3 | 25,0 | 0,0 | 100,0 | 75,0 | 83,3 | low | hd |
| Sparisoma rubripinne | 0,0 | 0,0 | 0,0 | 0,0 | 0,0 | 16,7 | low | hd |
| Sparisoma chrysopterum | 16,7 | 0,0 | 0,0 | 0,0 | 0,0 | 33,3 | low | hd |
| Sparisoma viride | 66,6 | 50,0 | 0,0 | 0,0 | 75,0 | 66,6 | low | hm |
| Thalassoma bifasciatum | 16,6 | 75,0 | 50,0 | 0,0 | 50,0 | 66,6 | low | im |
| Scarus vetula | 0,0 | 25,0 | 0,0 | 0,0 | 0,0 | 0,0 | low | hm |
| Lutjanus apodus | 0,0 | 0,0 | 0,0 | 0,0 | 25,0 | 16,7 | low | im, fc |
| Ocyurus chrysurus | 0,0 | 0,0 | 0,0 | 0,0 | 50,0 | 16,7 | low, high | fc |
| Lutjanus synagris | 0,0 | 0,0 | 0,0 | 0,0 | 0,0 | 16,7 | low | im |
| Aluterus scriptus | 0,0 | 0,0 | 0,0 | 0,0 | 25,0 | 0,0 | low, high | om |
| Mulloidichthys martinicus | 0,0 | 0,0 | 0,0 | 0,0 | 50,0 | 66,6 | low | im |
| Pseudupeneus maculatus | 0,0 | 0,0 | 0,0 | 0,0 | 0,0 | 50,0 | low | im |
| Gymnothorax moringa | 0,0 | 0,0 | 0,0 | 0,0 | 0,0 | 16,7 | bottom | fc |
| Ophichthus ophis | 0,0 | 0,0 | 0,0 | 0,0 | 0,0 | 16,7 | bottom | fc |
| Acanthostracion polygonium | 0,0 | 0,0 | 0,0 | 0,0 | 25,0 | 0,0 | low | is |
| Lactophrys triqueter | 0,0 | 0,0 | 0,0 | 0,0 | 0,0 | 16,7 | low | is |
| Holacanthus ciliaris | 0,0 | 0,0 | 0,0 | 0,0 | 25,0 | 0,0 | low | is |
| Holacanthus tricolor | 0,0 | 0,0 | 0,0 | 0,0 | 25,0 | 16,7 | low | is |
| Pomacanthus arcuatus | 0,0 | 0,0 | 0,0 | 0,0 | 50,0 | 0,0 | low | is |
| Pomacanthus paru | 0,0 | 0,0 | 0,0 | 0,0 | 25,0 | 0,0 | low | is |
| Abudefduf saxatilis | 0,0 | 25,0 | 50,0 | 0,0 | 50,0 | 33,3 | low, high | pk |
| Azurina cyanea | 33,3 | 0,0 | 0,0 | 100,0 | 0,0 | 33,3 | low, high | pk |
| Chromis insolata | 16,6 | 0,0 | 0,0 | 0,0 | 0,0 | 0,0 | low | pk |
| Microspathodon chrysurus | 33,3 | 0,0 | 0,0 | 0,0 | 0,0 | 33,3 | low | om |
| Stegastes adustus | 33,3 | 0,0 | 0,0 | 0,0 | 25,0 | 50,0 | low | om |
| Stegastes diencaeus | 0,0 | 0,0 | 0,0 | 0,0 | 25,0 | 0,0 | low | om |
| Stegastes leucostictus | 0,0 | 0,0 | 0,0 | 0,0 | 25,0 | 0,0 | low | om |
| Stegastes partitus | 33,3 | 0,0 | 0,0 | 100,0 | 25,0 | 83,3 | low | om |
| Stegastes planifrons | 100,0 | 100,0 | 100,0 | 100,0 | 75,0 | 100,0 | low | om |
| Azurina multilineata | 0,0 | 0,0 | 0,0 | 0,0 | 0,0 | 33,3 | low, high | pk |
| Heteropriacanthus cruentatus | 0,0 | 25,0 | 0,0 | 0,0 | 50,0 | 0,0 | low, high | pk |
| Odontoscion dentex | 66,6 | 0,0 | 0,0 | 0,0 | 50,0 | 0,0 | low | im |
| Eques punctatus | 16,6 | 0,0 | 0,0 | 0,0 | 50,0 | 0,0 | low | im |
| Scorpaena plumierii | 0,0 | 0,0 | 0,0 | 0,0 | 0,0 | 16,7 | bottom | fc |
| Cephalopholis cruentata | 50,0 | 25,0 | 50,0 | 100,0 | 50,0 | 66,6 | low | im |
| Cephalopholis fulva | 0,0 | 0,0 | 0,0 | 0,0 | 0,0 | 16,7 | low | im |
| Hypoplectrus nigricans | 16,6 | 0,0 | 0,0 | 0,0 | 25,0 | 0,0 | low | im |
| Hypoplectrus puella | 33,3 | 25,0 | 50,0 | 100,0 | 100,0 | 50,0 | low | im |
| Hypoplectrus randallorum | 0,0 | 0,0 | 0,0 | 0,0 | 50,0 | 0,0 | low | im |
| Hypoplectrus unicolor | 0,0 | 0,0 | 0,0 | 0,0 | 50,0 | 0,0 | low | im |
| Serranus tigrinus | 0,0 | 25,0 | 0,0 | 0,0 | 25,0 | 33,3 | low | im |
| Synodus intermedius | 0,0 | 0,0 | 0,0 | 0,0 | 25,0 | 50,0 | bottom | fc |
| Synodus synodus | 0,0 | 0,0 | 0,0 | 0,0 | 0,0 | 16,7 | bottom | fc |
| Canthigaster rostrata | 33,3 | 0,0 | 50,0 | 100,0 | 50,0 | 83,3 | low | om |
| Sphoeroides spengleri | 0,0 | 0,0 | 0,0 | 0,0 | 0,0 | 33,3 | low | om |
